# Brain Herniation Through the Cribriform Plate: Review and Comparison to Encephaloceles in the Same Region

**DOI:** 10.7759/cureus.2961

**Published:** 2018-07-10

**Authors:** Rabjot Rai, Joe Iwanaga, Marios Loukas, Rod J Oskouian, R. Shane Tubbs

**Affiliations:** 1 Anatomy, St. George's University School of Medicine, St. George's, GRD; 2 Medical Education and Simulation, Seattle Science Foundation, Seattle, USA; 3 Anatomical Sciences, St. George's University, St. George, GRD; 4 Neurosurgery, Swedish Neuroscience Institute, Seattle, USA; 5 Neurosurgery, Seattle Science Foundation, Seattle, USA

**Keywords:** cribriform plate, herniation, nasal encephalocele, cerebrospinal fluid (csf) fistula

## Abstract

Herniations of the brain and/or meninges through an opening of the skull often occur through the foramen magnum, e.g., Chiari malformations and encephaloceles. The herniation of brain matter through the cribriform plate is a rare incident and has not been reported frequently. The presence of such an occurrence still requires attention and anatomical understanding. This review will examine the potential causes of cribriform plate herniation and its distinguishability to nasal encephaloceles. The sloping of brain tissue toward potential space/opening in response to elevated pressures in the cranium to accommodate for the added pressure are features seen in herniation. The presence of a pedicle and stalk seen in an encephalocele define its characteristics, which are not visible in a ‘classical’ herniation. Cerebrospinal fluid (CSF) fistula commonly occurs at the cribriform plate, and due to the structural weakness, a pathway is formed. This is often seen in conjunction with meningoceles. Delineating between herniation and encephaloceles is important for both clinicians and neurosurgeons.

## Introduction and background

Brain herniation describes the protrusion of brain tissue through an opening through the skull often when the fixed volume of the cranium is unable to compensate for an increase in intracranial pressure (ICP). The increases in pressure are commonly due to space-occupying lesions, such as tumors, hemorrhage, cerebral edema, or inflammation. Normally, the brain will try to counteract the added volume by decreasing volume elsewhere in order to maintain equilibrium; shifting CSF or reducing venous blood flow may accomplish this, i.e., the Monro-Kellie doctrine. The herniation of brain tissue is more commonly observed traversing the foramen magnum, tentorial incisura, and subfalcine space [1-2].

Reports of herniation through the cribriform plate are rare. This review will differentiate between herniation and encephalocele at the cribriform plate.

## Review

Anatomy

The cribriform plate, a horizontal segment of the ethmoid bone, forms a major portion of the nasal roof separating it from the anterior cranial fossa. Cribriform is derived from the Latin term, cribrum, meaning sieve, signifying the numerous foramina found in the structure of the cribriform plate. The foramina allow the olfactory nerves to course through toward the olfactory bulb [3-5]. The lateral laminae of the cribriform plate are the thinnest portions of the ethmoid roof, making the cribriform structure anatomically delicate to increased intracranial pressure [2, 6].

Etiology

**Figure 1:**
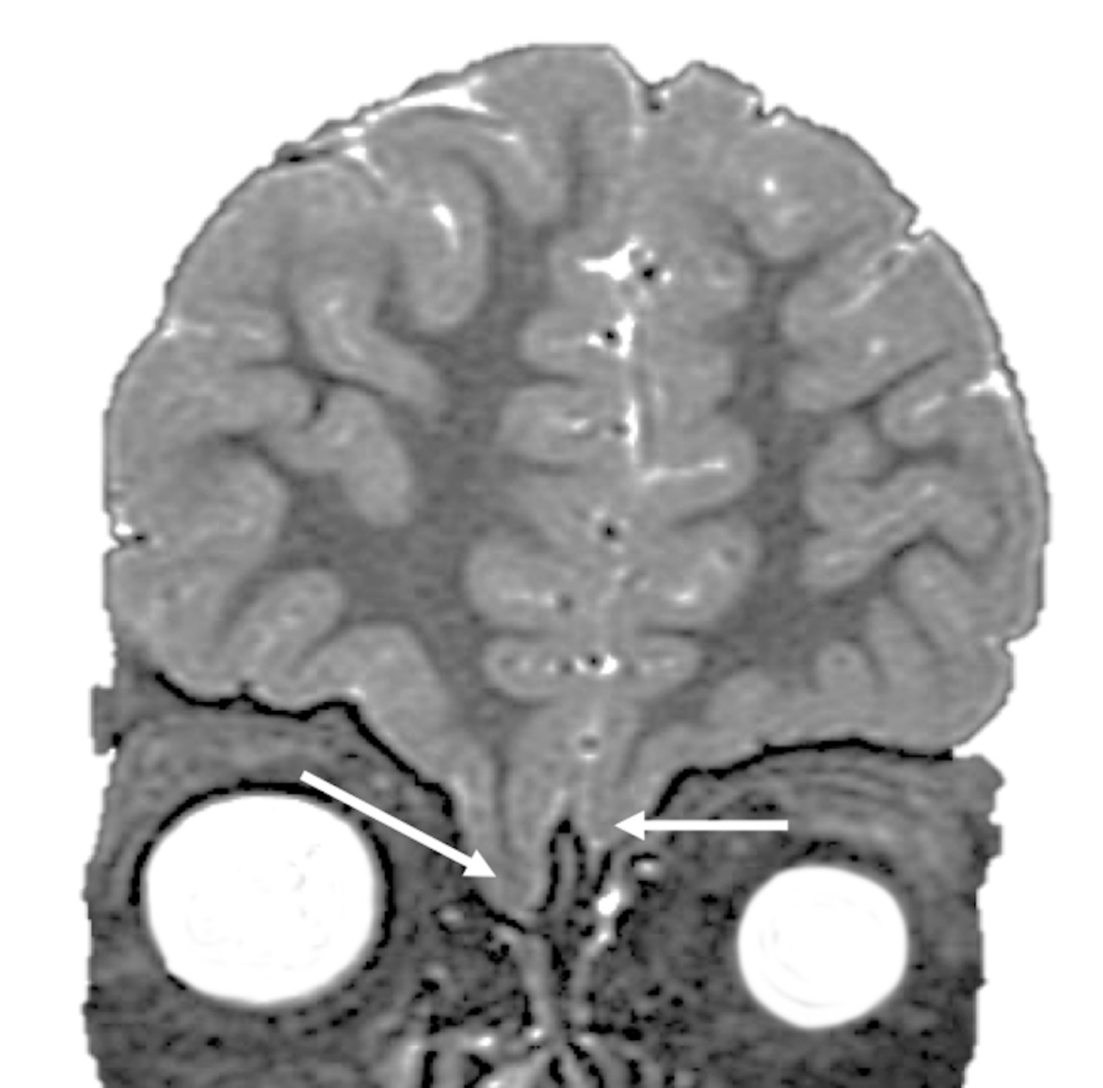
Coronal MRI illustrating extensive downward sloping of brain tissue (frontal lobes) through the cribriform plate Note the brain tissue on the right side is more herniated when compared with the left side, which is also herniated (arrows). MRI: magnetic resonance imaging

Herniation through structural openings of the cribriform plate is viewed more so as a downward sloping of tissue matter comprised of brain and/or meningeal tissue (Figure 1). The causes of herniation can be congenital or acquired. Congenital herniation is thought to be due to an error with the development of the mesoderm, whereas acquired forms of brain herniation are secondary to traumatic or iatrogenic origins. Herniation through the anterior cranial fossa and through the cribriform plate requires rigid reconstruction with a multilayer repair being necessary in these patients in order to avoid spinal fluid leakage [2].

Theories

A theory suggested by Dange et al. proposed that chronically sustained intracranial hypertension upon the cribriform plate resulted in erosion [2]. Their study presented the case of a space-occupying lesion (an extra-axial mass) which resulted in the progressive destruction of the cribriform plate secondary to increased intracranial pressure. Dange et al. suggested that the compensatory measures to balance the increased pressure proceeded to allow the herniation of brain tissue through the cribriform plate. Another possibility proposed by Dange et al. was that the presence of a congenital small dehiscence on the cranial floor had enlarged during the herniation of the brain tissue, leading to herniation through the cribriform plate and into the nasal cavity. Anatomical variations observed in the cribriform plate may also lead to the plate becoming weakened, thus allowing herniation to follow. These variations include a lack of occlusion of the foramina along the olfactory fibers in the cribriform plate, a persistent cranial pharyngeal canal, the presence of a fistula in the embryological remnants of the olfactory bulb lumen, and meningeal dysplasia at the site of the olfactory nerves [2].

Comparison to encephaloceles

Delineating between herniation and encephalocele is not clear-cut; there are apparent overlaps. However, this review will outline key defining differences. Encephalocele is the herniation of a sac appearing as a pedicle with a stalk, containing meninges and/or brain tissue [7]. By taking the path of least resistance, encephaloceles can protrude through weakened bony structures of the skull [8]. The dura mater surrounding encephaloceles is described as being thinner and irregular compared to the normal dura mater. The size of the encephalocele is important in terms of the surgical approach taken—smaller sacs are invaginated and the dura mater is sutured, while larger sacs are excised. In order to excise the encephalocele, the neck of the sac needs to be secured and in order to allow access to this area, a craniotomy is performed [9].

An encephalocele along and near the cribriform plate is known as a nasal encephalocele and these are classified as basal and frontoethmoidal (or sincipital) encephaloceles [7, 9]. Basal encephaloceles make up 40% of all nasal encephaloceles and may be clinically undetected for years as they present in the nasal cavity [8, 10]. Basal encephaloceles are further classified into 1) transethmoidal - a sac protruding through a defect in the cribriform plate into the superior meatus and appearing as a nasal polyp, 2) sphenoethmoidal - a sac herniating through the cribriform plate between the posterior ethmoidal cells and sphenoidal sinus into the nasopharynx, 3) spheno-orbital - a sac protruding through the superior orbital fissure into the orbit, and lastly, 4) transsphenoidal - a defect in the posterior cribriform plate leading to a sac herniating into the nasopharynx [10]. Frontoethmoidal encephaloceles (found in 60% of all nasal encephaloceles) appear as a soft, compressible, external mass over the glabella [8, 10]. These are subtyped into nasofrontal, nasoethmoidal, and naso-orbital types [10]. More importantly, nasal encephaloceles arise as a result of ‘late’ neurulation defects in the fourth week of gestation and are thought to occur due to the failure of the neuroectoderm (nervous tissue) to separate from the surface ectoderm (epithelial layer) [8, 11].

Complications

The cribriform plate is vulnerable to CSF leaks due to the thin nature of the bone, creating a structural weakness and forming a pathway for potentials leaks. These patients may present with CSF rhinorrhea or meningitis. With a spontaneous CSF fistula, there is a high rate of a coexisting meningoencephalocele, which are often found incidentally during magnetic resonance imaging (MRI) imaging when evaluating CSF leaks. These fistulas are more common in middle-aged obese females, typically fitting the signs and symptoms similar to idiopathic intracranial hypertension (IIH). Other pathogeneses include abnormal absorption of CSF by arachnoid granulations, empty sella syndrome, and destruction of olfactory components in the cribriform plate or pituitary gland allowing for extra space for the extension of the arachnoid space [12]. Alonso et al. proposed that the occurrence of CSF fistula is multifactorial with a combination of both increased intracranial pressure with atrophy of the cranial base (pneumatization of bone, thinning cortical bone, and progressive destruction) [2, 12].

A thorough knowledge of the normal anatomical structures and variations of the regions surrounding the cribriform plate are necessary for a thorough understanding of cribriform plate herniations [13-17].

## Conclusions

Herniation through the cribriform plate may be due to the congenital weakening of it or may be secondary to increased intracranial pressure. Clinicians and neurosurgeons should be aware of the similarities and differences between these pathological entities and encephaloceles.
